# Retained Metallic Intraocular Foreign Body With Excellent Visual Outcome

**DOI:** 10.7759/cureus.18633

**Published:** 2021-10-09

**Authors:** Poh Fong She, Evelyn Tai, Akmal Haliza Zamli, Rohana Abdul Rashid, Safinaz Mohd Khialdin

**Affiliations:** 1 Ophthalmology, Universiti Kebangsaan Malaysia Medical Centre, Kuala Lumpur, MYS; 2 Ophthalmology, Hospital Tengku Ampuan Afzan, Kuantan, MYS; 3 Ophthalmology, School of Medical Sciences, Universiti Sains Malaysia, Kubang Kerian, MYS

**Keywords:** endophthalmitis, siderosis bulbi, metallic foreign body, penetrating ocular injuries, intraocular foreign body

## Abstract

A healthy 25-year-old gentleman sustained a left eye perforating injury involving a metallic intraocular foreign body. Upon examination, his best-corrected visual acuity was 6/6 in the right eye and 1/60 in the left eye. There was a full thickness cornea-scleral laceration wound with uveal tissue prolapse at 7 o’clock. The pupil was peaked inferonasally. The anterior chamber was deep with cells grade 4+ and a hyphema level. The posterior segment could not be visualized due to a vitreous haemorrhage. The computed tomography scan revealed a high-density foreign body embedded in the posterior wall of the globe. He underwent primary toilet and suturing of the left eye cornea-scleral laceration, followed by pars plana vitrectomy with an endolaser and gas tamponade. However, the foreign body could not be identified intraoperatively. Post-operation, the left eye vision improved, achieving his premorbid best-corrected visual acuity of 6/6 by six months post-op. The intraocular foreign body was managed conservatively in view of the excellent visual acuity and the risk of further surgery. The patient has remained asymptomatic since then until his last follow-up at 30 months post-operation.

## Introduction

Ocular trauma is a leading cause of preventable monocular blindness among young males [1]. It is classified by the Birmingham Eye Trauma Terminology system into open and closed globe injuries [2]. An open globe injury may either be a rupture or a laceration. Lacerations include penetrating injuries (entry wound only), perforating injuries (with an entry and an exit wound), or injuries associated with intraocular foreign bodies (IOFB). Open globe injuries are relatively rare, comprising 1-2% of ocular trauma [3]. These injuries tend to have a worse visual prognosis than closed globe injuries, particularly in the presence of IOFB [4]. We present a case of a patient who maintained excellent vision despite a penetrating eye injury involving a retained metallic IOFB.

## Case presentation

A healthy 25-year-old gentleman presented to the ophthalmology clinic complaining of sudden onset of left eye pain and blurring of vision. He was watching his colleague hammering metal when he felt something strike his left eye with high velocity. He was not wearing safety goggles at the time.

**Figure 1:**
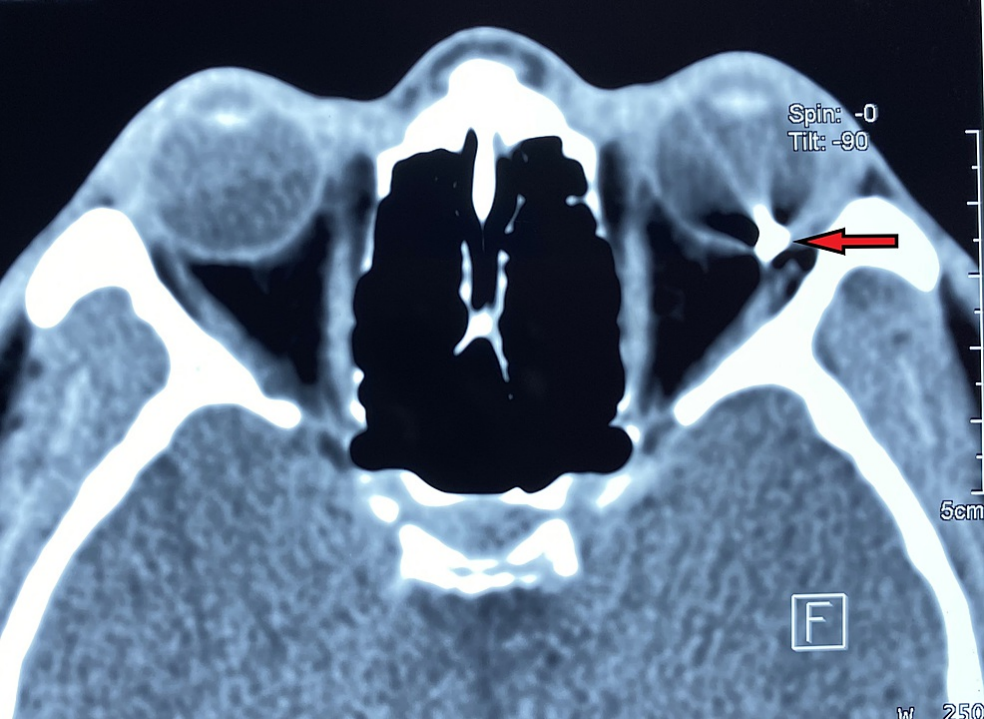
CT imaging demonstrating a foreign body (arrow)

Upon examination, his best-corrected visual acuity was 6/6 in the right eye and 1/60 in the left eye. There was a full thickness cornea-scleral laceration wound with uveal tissue prolapse at 7 o’clock. The pupil was peaked inferonasally. The anterior chamber was deep with cell reaction of grade 4+ and a hyphema level measuring 2mm vertically. The lens and iris were normal. Extraocular muscle movement was full. The relative afferent pupillary defect was negative. The posterior segment could not be visualized due to the hazy media. B scan ultrasound showed a vitreous haemorrhage. Computed tomography of the orbits revealed a high-density foreign body embedded in the posterior wall of the globe measuring 0.8 cm in height and 0.4 cm in the anteroposterior diameter (Figure 1). The right eye was normal.

He underwent toilet and suturing of the left eye cornea-scleral laceration wound with intravitreal injection of vancomycin and ceftazidime. He was also commenced on intravenous ciprofloxacin. A week later, a left pars plana vitrectomy with an endolaser and gas tamponade was performed. However, the foreign body was not identified intraoperatively.

**Figure 2:**
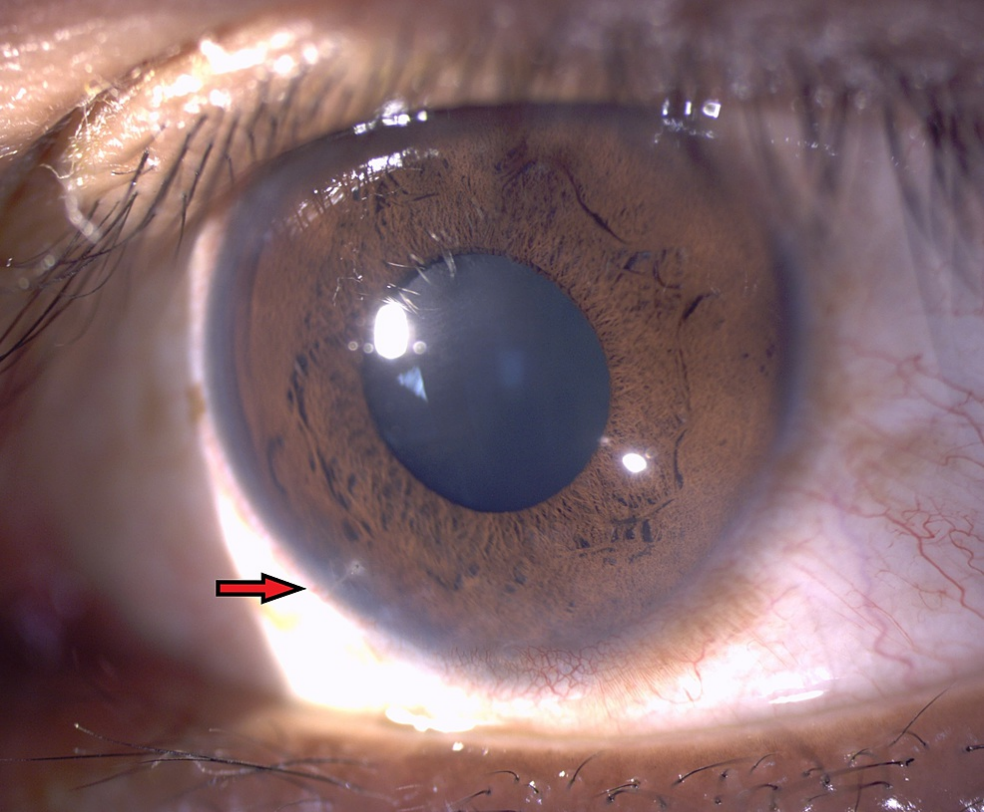
Left eye anterior segment photo demonstrating a peripheral corneoscleral scar at 7 o’clock denoting the entry wound of the intraocular foreign body

**Figure 3:**
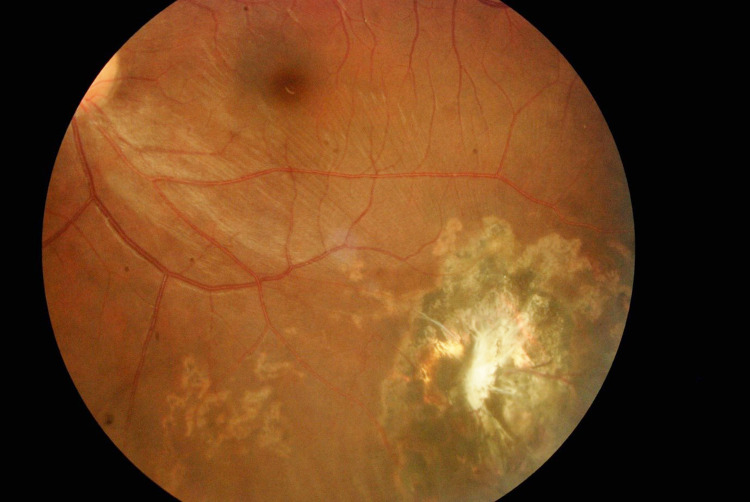
Left eye fundus photo shows a chorioretinal scar over the inferotemporal quadrant of the retina

Post-operation, the left eye vision improved, achieving his premorbid best-corrected visual acuity of 6/6 by six months post-op. The left eye healed with a peripheral corneoscleral scar (Figure 2) and inferotemporal chorioretinal scar (Figure 3). The IOFB was managed conservatively in view of the excellent visual acuity. The patient has remained asymptomatic since then until his last follow-up at 30 months post-operation.

## Discussion

Occupational eye injuries are a significant cause of acquired visual disability, particularly in developing countries [2]. The most frequent mechanism of eye trauma is sharp injury (82.4%), followed by IOFB, blunt injury, and blast injury [5]. Approximately 75% of IOFB involve the posterior segment [6]. The majority are metallic, acquired by hammering, usually in the absence of protective eyewear [7].

According to the International Ocular Trauma Score, factors affecting the visual outcome in ocular trauma include initial visual acuity, globe rupture, endophthalmitis, perforating injury, retinal detachment, and the presence of a relative afferent pupillary defect [8]. In cases compounded by IOFBs, the extent of intraocular damage is related to the mode of injury, nature of the IOFB and associated complications such as vitreous loss and retinal damage [9]. In addition, a sharp IOFB tends to cause less damage than an equivalent blunt one, presumably because damage from sharp objects is confined to the involved tissue while blunt IOFB are associated with a higher energy transfer to the eye at the time of impact [6,10].

Approximately 50% of IOFB can only be identified via computed tomography imaging [1]. The main complications of a retained metallic IOFB include endophthalmitis (16.76%) and siderosis bulbi (2.74%), which may develop from days to years post-trauma [6,11]. However, not all cases of iron IOFB result in siderosis bulbi. Arnaiz et al. suggested that the reason for prolonged asymptomatic retained IOFBs may be its encapsulation by a thin membrane in the area [12]. Endophthalmitis has been reported more than a decade after ocular trauma with retained IOFB [13-14]. This indicates the need for long-term follow-up in cases of incarcerated IOFB. An electroretinogram and visual field test can be used to assess the retinal dysfunction in patients with IOFB.

Our case illustrates a rare presentation in which the patient had an excellent visual outcome despite an incarcerated metallic IOFB. Conservative management is an option in cases where there are no signs of infection and the risk of surgery outweighs its benefits [9]. The risk of endophthalmitis in these cases is generally low, especially with the use of broad-spectrum topical and systemic antibiotics [1,9].

## Conclusions

Conservative management may be the only avenue when other treatment modalities have failed to remove an IOFB. Vision can remain good even in cases of a retained metallic IOFB, although lifelong follow-up is required. Education and legislation on eye safety, especially mandating the use of protective eyewear during high-risk activities, may reduce the incidence of ocular trauma.
